# Abnormal expression of TBX4 during anorectal development in rat embryos with ethylenethiourea-induced anorectal malformations

**DOI:** 10.1186/s40659-019-0235-6

**Published:** 2019-05-04

**Authors:** Meng Li, Hailan Zhang, Huiying Liu, Hongzhong Tian, Xiaobing Tang, Yuzuo Bai, Weilin Wang

**Affiliations:** Department of Pediatric Surgery, Shengjing Hospital of China Medical University, No. 36 SanHao St., Heping District, Shenyang, 110004 China

**Keywords:** Anorectal malformations, TBX4, Embryogenesis

## Abstract

**Background:**

To assess the expression of T-box transcription factor 4 (TBX4) during the anorectal development in normal and ethylenethiourea (ETU)-induced anorectal malformations (ARM) rat embryos.

**Methods:**

Anorectal malformations was induced by ETU on the 10th gestational day (E10) in rat embryos. Spatio-temporal expression of TBX4 was evaluated in normal (n = 490) and ETU-induced ARM rat embryos (n = 455) from E13 to E16 by immunohistochemical staining, Western blot analysis and real-time RT-PCR.

**Results:**

In the normal embryos, immunohistochemical staining revealed that TBX4 expression was detected in the epithelium of hindgut and urorectal septum (URS) on E13. TBX4-immunopositive cells were increased significantly in the epithelium of hindgut and URS, the future anal orifice part of cloacal membrane on E14. On E15, abundant stained cells were observed in the rectum, URS and dorsal cloacal membrane and the expression of positive cells reached its peak. On E16, only sporadic positive cells were distributed in the epithelium of the distal rectum. In the ARM embryos, the hindgut/rectum, URS and dorsal cloacal membrane were faint for TBX4 immunohistochemical staining. In the normal group, TBX4 protein and mRNA expression showed time-dependent changes in the hindgut/rectum from E13 to E16 on Western blot and real-time RT-PCR. On E13 and E15, the expression level of TBX4 mRNA in the ARM group was significantly lower than that in the normal group (*P *< 0.05). On E15, the expression level of TBX4 protein in the ARM group was significantly lower than that in the normal group (*P *< 0.05).

**Conclusions:**

The expression of TBX4 was downregulated in ETU-induced ARM embryos, which may play important roles in the pathogenesis of anorectal development.

## Background

Anorectal malformations (ARM) constitute a significant proportion of congenital gastrointestinal defects. In the world, the incidence of ARM is quoted as 2–6 per 10,000 live births, which changes in the reports of different countries and years [1, 2]. The clinical manifestations of ARM are different, may appear alone, may be combined with other abnormalities, or as part of the syndrome [3]. Although newborn care and surgical techniques progress, many ARM patients still need to accept challenges for a long time including the bowel and bladder dysfunction, sexual dysfunction, and social psychological problems [4, 5]. Epidemiological and genetic studies have shown that the occurrence of ARM is associated with environmental factors and genetic factors [6].

Molecular biology studies showed that the occurrence of ARM was related to many genes, especially the Hox gene family, sonic hedgehog (SHH) signaling pathway, bone morphogenetic protein 4 (BMP4) gene and fibroblast growth factor 10 (FGF10) gene [7–10]. T-box transcription factor 4 (TBX4) is a known upstream regulator of FGF10 [11, 12]. TBX4 was expressed in the epithelium of the gastrointestinal tract in a manner similar to SHH [13]. However, the expression of TBX4 has not been described in normal anorectal development and ARM ever before. In order to reveal the regulation effect of TBX4 on the development of anorectum and ARM, we analyzed the distribution and the expression level of TBX4 from E13 to E16 in rat embryos with ethylenethiourea (ETU)-induced ARM.

## Methods

### Sample collection and preparation

The study was approved by the ethics committee of Shengjing Hospital Affiliated to China Medical University. The Wistar rats were mated for the night, and the next morning the female rats were given vaginal smear. When the sperm was seen under the microscope, it was suggested to mate, and the day was 0 day (E0). The pregnant rats were randomly divided into two groups. The ETU group: 40 pregnant rats were given 1% ETU solution by gavage on E10 and continued to maintain pregnancy. The normal group: 40 pregnant rats were given normal saline on E10. A certain number of pregnant rats in the normal and ETU groups were chosen according to the plan from E13 to E16. The pregnant rats were treated with 10% chloral hydrate (4 mL/kg) by intraperitoneal injection. All fetal rats were removed and the whole embryos were taken. A part of the specimens were fixed in 4% paraformaldehyde phosphate buffer and embedded in paraffin. The wax blocks were continuously sliced in the middle sagittal plane, and the slice thickness was 4 μm. The sections were divided into normal group and ETU-induced ARM group in preparation for immunohistochemical staining. Other specimens were frozen in liquid nitrogen. The sagittal sequential sections were examined to confirm with or without ARM of ETU-treated embryos under the light microscope. Under the dissecting microscope (Olympus Szhillb, Japan), the hindgut was dissected and removed from the surrounding tissues on E13 and E14, and a full-thickness rectum was dissected on E15 and E16 to prepare for Western Blot and RT-PCR in the normal and ARM embryos.

### Immunohistochemical staining

Immunohistochemistry (IHC) analysis of midsagittal sections was performed to analyze the spatiotemporal expression of TBX4 in the cloaca of rat embryos. The tissue sections were first incubated in 3% H_2_O_2_ for 20 min to block endogenous peroxidase activity. The non-specific antigen of the sections was sealed with 10% fetal bovine serum. The sections were incubated with TBX4 antibody (1:300 goat polyclonal, sc-17881, Santa Cruz, Europe) at 4 °C for 12 h. The next morning, the sections were incubated at room temperature for 45 min. The incubation of polymer adjuvant was performed for 20 min. The sections were incubated in horseradish peroxidase (HRP)-labeled rabbit anti-goat IgG polymer (Polink-2 plus polymer HRP Detection System, ZSGB-BIO, China) for 20 min at room temperature. Signals were visualized using freshly prepared 3′ 3 diaminobenzidine. Sections were counterstained with hematoxylin.

### Protein preparation and western blot

Nuclear protein was isolated using the Minute Cytosolic and Nuclear Extraction kit (SC-003, Invent Biotechnologies, Eden Prairie, MN) according to the manufacturer’s instructions. Tissues collected from the hindgut or rectum of ARM model and normal rat embryos were pooled and sonicated in double-distilled water containing protease inhibitors. Protein extracts (100 μg) were denatured by heating at 95 °C for 5 min, and then stored at − 80 °C refrigerator until used. Protein samples were separated by sodium dodecyl sulfate-polyacrylamide gel electrophoresis (SDS-PAGE, Beyotime, Shanghai, China), transferred to the polyvinylidene fluoride (PVDF) membranes (Millipore, Billerica, MA, USA), blocked with 5% fat-free milk in Tris-buffered saline for 2 h at room temperature, and incubated overnight at 4 °C with TBX4 goat polyclonal antibody (diluted 1:1000, sc-17881, Santa Cruz, Europe) or Histone-H3 rabbit polyclonal antibody (diluted 1:2000, 17168-1-AP, Proteintech, USA) respectively. The membranes were incubated with secondary antibody [diluted 1:2000, peroxidase-conjugated donkey anti-goat (A0181, Beyotime, Beijing, China) or goat anti-rabbit IgG (ZB-2301, ZSGB-BIO, Beijing, China)] respectively for 2 h at room temperature. A chemiluminescent substrate kit (Pierce, Rockford, IL, USA) was used to detect the immunostained bands. In each lane, Histone-H3 was used as an internal standard to normalize protein expression.

### RNA isolation and real-time RT-PCR

Total RNA was isolated with the TRIzol reagent (Invitrogen Life Technologies, Carlsbad, CA, USA) according to the manufacturer’s protocol. The purity of extracted total RNA was determined by the 260:280 nm ratio with an expected value of 1.8 to 2.0. RNA (1 μg) was reverse-transcribed into complementary DNA (cDNA) by using the PrimeScript RT reagent kit (Takara Biotechnology, Shiga, Japan) following the manufacturer’s instructions. Quantitative real-time RT-PCR was accomplished with SYBR Premix Ex Taq Kit (Takara Biotechnology, Shiga, Japan) on the 7500 real-time PCR system (Applied Biosystems) under the following conditions: 95 °C for 5 min, 45 cycles of 95 °C for 15 s, 60 °C for 45 s. A dissociation procedure was performed to generate a melting curve for confirmation of amplification specificity. β-actin was used as the reference gene. The relative levels of gene expression were represented as ∆Ct = Ct gene − Ct reference, and the fold change in gene expression was calculated with the comparative Ct (2−∆∆Ct) method [14]. Experiments were repeated in triplicate. The primer sequences spanning the intron–exon junction were as follows: TBX4 forward, 5′-CCTTCCCCACACACTTCACC-3′; reverse, 5′-AGGGTGGCTTCCTCTCACAC-3′; β-actin forward, 5′-GGAGATTACTGCCCTGGCTCCTA-3′; reverse, 5′- GACTCATCGTACTCCTGCTTGCTG-3′.

### Statistical analysis

All numerical data are presented as mean ± SD. Statistical analysis was performed by using the independent sample t test. *P *< 0.05 was considered significant.

## Results

### General observations

No malformations were observed in the 490 embryos of the normal rats. However, all ETU-treated embryos had a short tail or no tail. The incidence of ARM in the ETU-treated embryos was 80%. The type of ARM was rectourethral fistula or persistent cloaca. The distribution of embryos in the normal and ARM groups were shown in Table 1.

**Table 1:** Distribution of embryos in the normal and ARM groups

Age	Normal embryos			ARM embryos		
	IHC	WB	PCR	IHC	WB	PCR
E13	25	70	71	24	67	68
E14	25	59	59	27	55	56
E15	26	33	37	21	31	34
E16	12	37	36	11	30	31
Total	88	199	203	83	183	189

*E* embryonic day, *ARM* anorectal malformations, *IHC* immunohistochemical staining, *WB* western blot, *RT-PCR* real-time quantitative polymerase chain reaction

### IHC results

#### Normal group

**Fig. 1:**
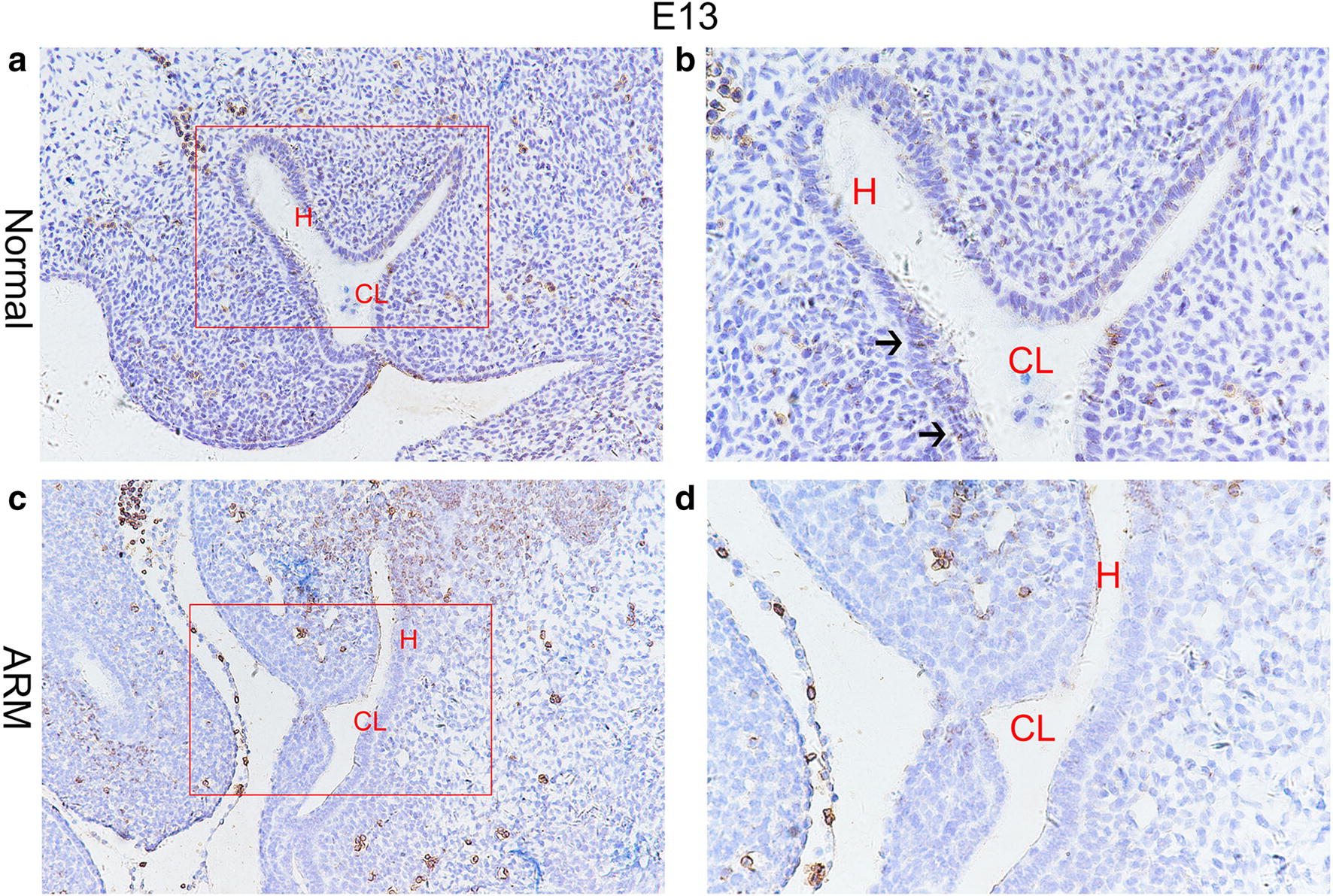
On E13. TBX4-immunopositive cells were distributed in the epithelium of the hindgut and urorectal septum in normal group (**a**, **b**). No positive cells were seen in the hindgut or urorectal septum in ARM group (**c**, **d**). Red rectangles in **a** and **c** are shown at higher magnification in **b** and **d**. ×200 (**a**, **c**). ×400 (**b**, **d**). Black arrowheads: positive cells. *CL* Cloaca, *H* hindgut

**Fig. 2:**
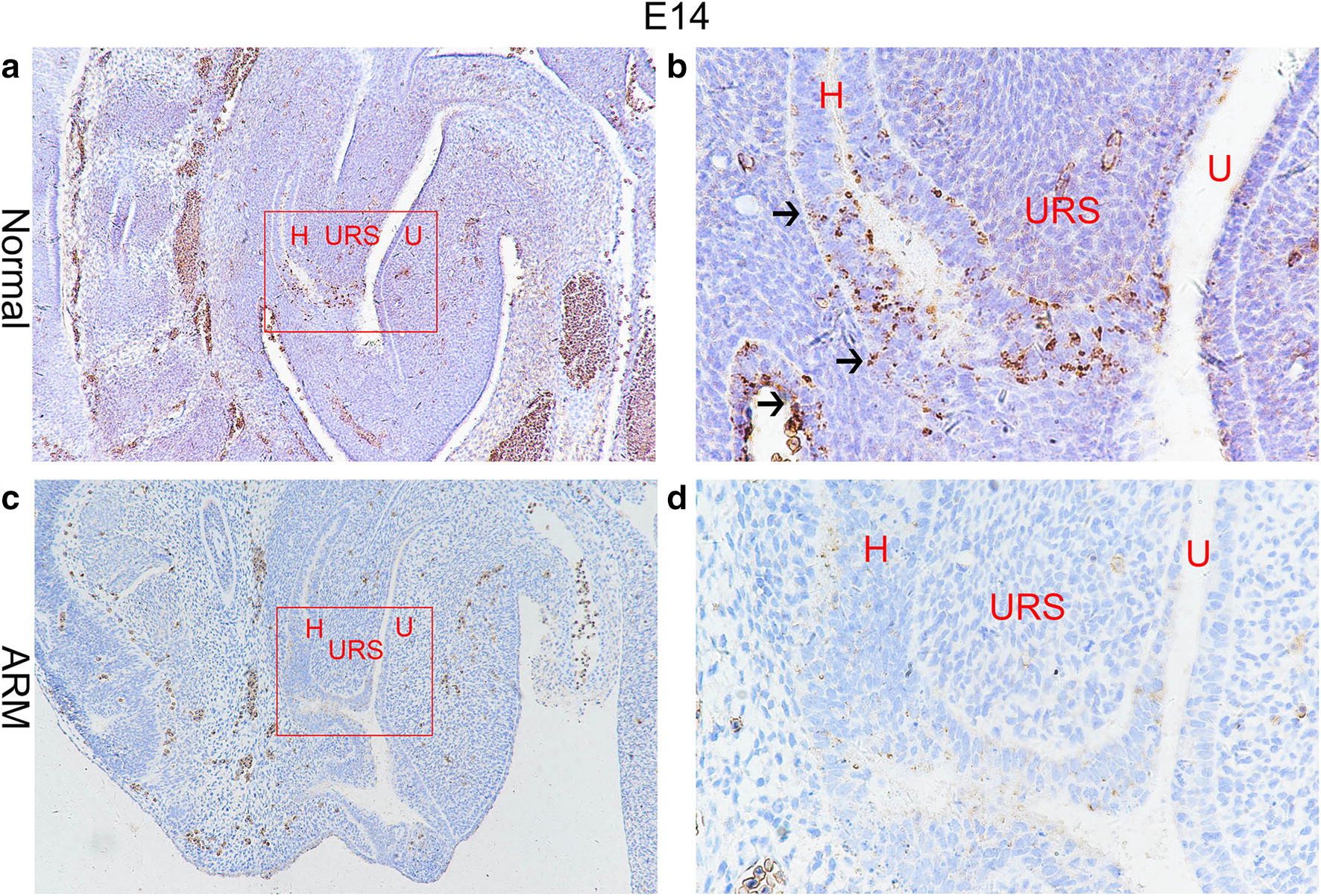
On E14. TBX4-immunopositive cells were increased significantly in the epithelium of hindgut and URS, future anal orifice part of the cloacal membrane in normal group (**a**, **b**). The TBX4-immunopositive cells were scattered in the epithelium of the hindgut and dorsal cloacal membrane in ARM group (**c**, **d**). Red rectangles in **a** and **c** are shown at higher magnification in **b** and **d**. ×100 (**c**, **d**). ×400 (**b**, **d**). Black arrowheads: positive cells. *H* hindgut, *U* urogenital sinus, *URS* urorectal septum

**Fig. 3:**
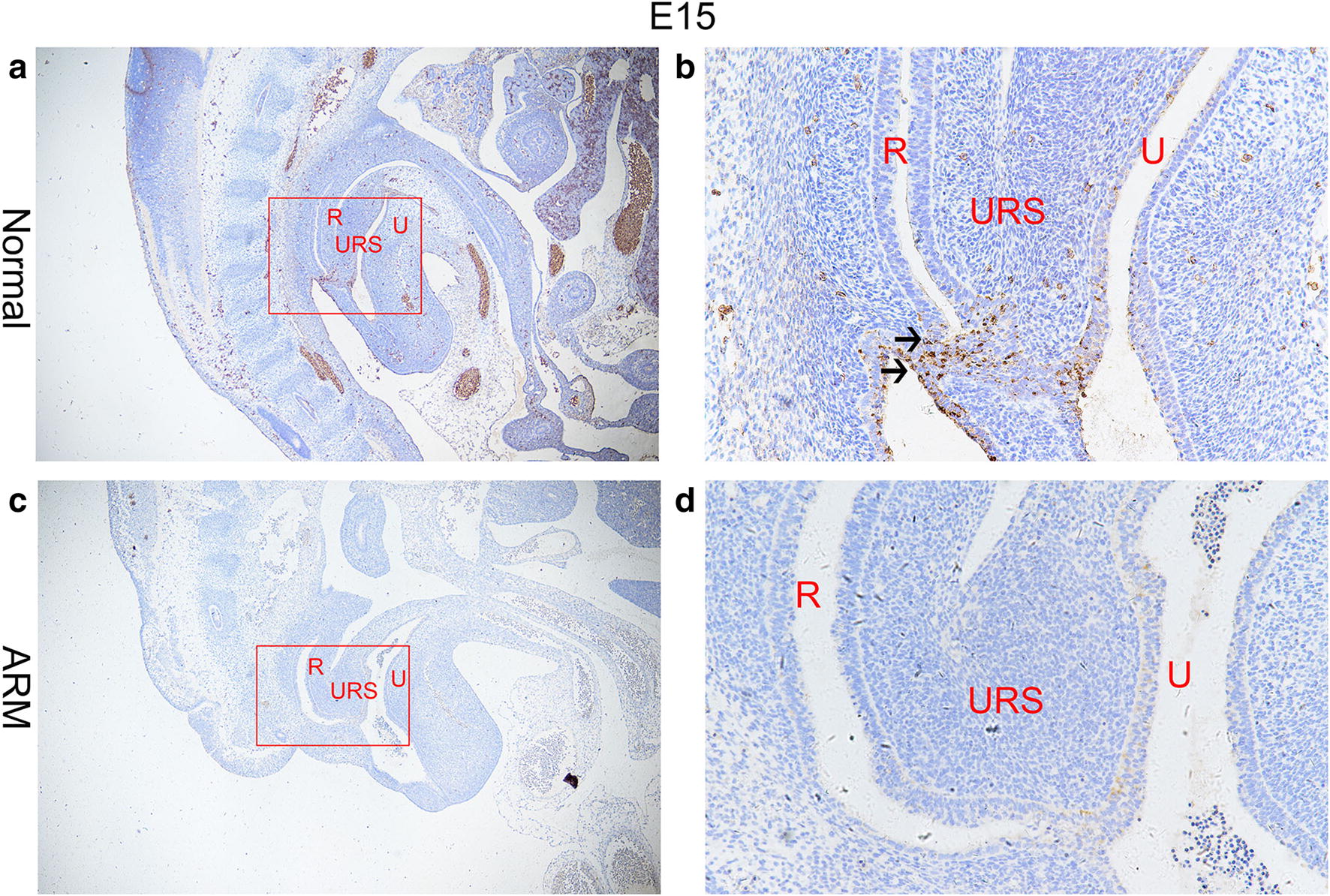
On E15. A large number of TBX4-immunopositive cells were distributed in the epithelium of the rectum and URS, and the anal membrane in normal group (**a**, **b**). A fistula was seen between the rectum and urethra in ARM group. The expression of TBX4 in anal membrane and the epithelium of the rectum were very weak (**c**, **d**). Red rectangles in **a** and **c** are shown at higher magnification in **b** and **d**. ×100 (**a**, **c**). ×400 (**b**, **d**). Black arrowheads: positive cells. *URS* urorectal septum, *U* urethra, *R* rectum

**Fig. 4:**
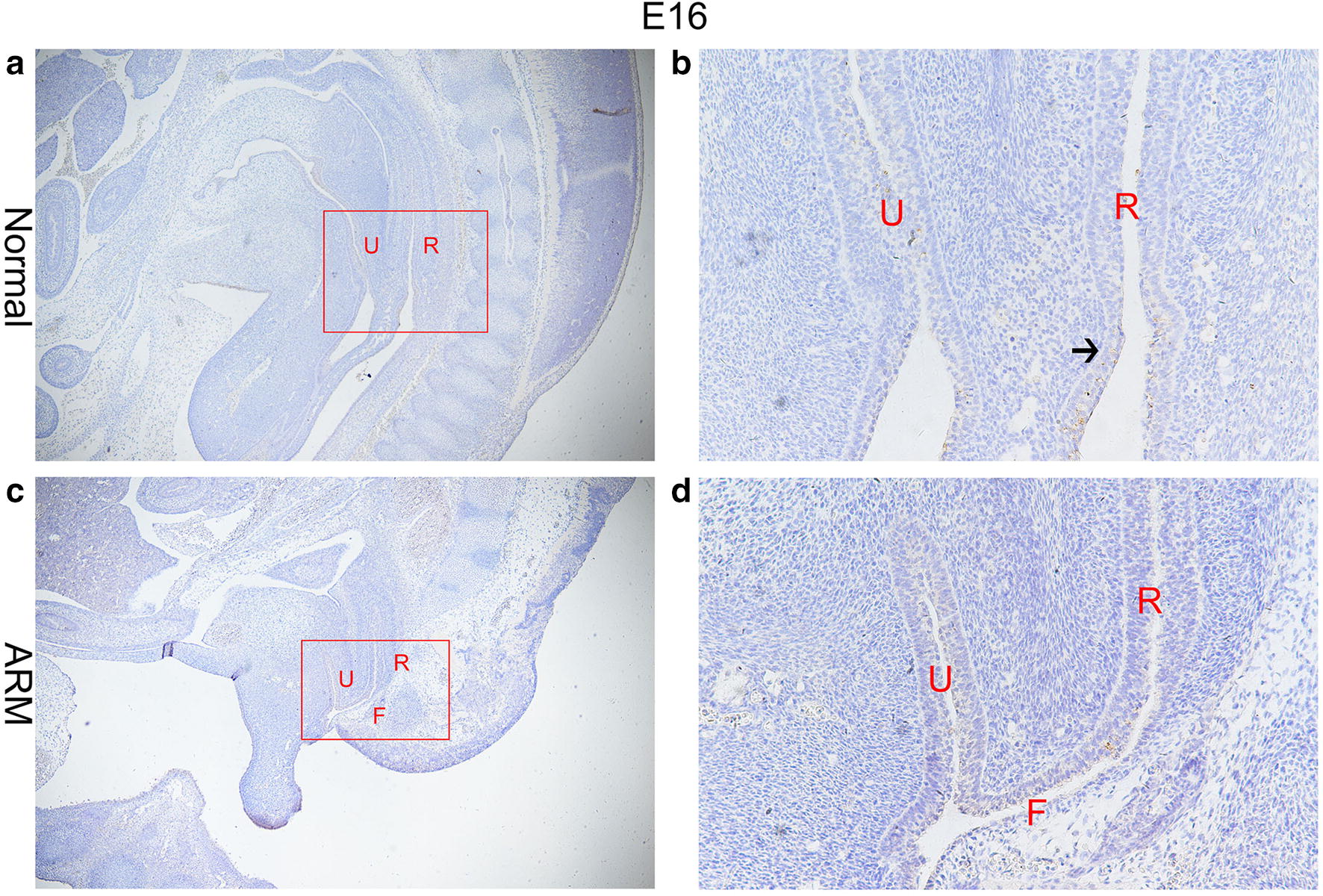
On E16. The anal membrane ruptured and rectum communicated with the outside. Rare TBX4 positive cells were expressed in the epithelium of the distal rectum in normal group (**a**, **b**). The fistula between the urethra and rectum of the ARM embryos still existed, and only sporadic TBX4-immunopositive cells were distributed in the epithelium of the rectum and the fistula (**c**, **d**). Red rectangles in **a** and **c** are shown at higher magnification in **b** and **d** ×40 (**a**). ×100 (**c**). ×400 (**b**, **d**). Black arrowhead: positive cells. *F* Fistula, *U* urethra, *R* rectum

On E13, the cloaca had formed at the end of the tail, and the urorectal septum (URS) divided the cloaca into the urogenital sinus (UGS) and the primitive rectum (hindgut). TBX4-immunopositive cells were distributed in the epithelium of the hindgut and URS. The specific staining was subtle (Fig. 1a, b). On E14, the URS descended and divided clearly the cloaca into the UGS and hindgut, and TBX4-immunopositive cells in the epithelium of hindgut and URS increased significantly. TBX4-immunopositive cells were abundantly observed in the future anal orifice part of cloacal membrane (Fig. 2a, b). On E15, the epithelium on the tip of the URS fused with that of the dorsal cloacal membrane leading to the separation of the rectum and urethra. The anal membrane was thin. Immunohistochemical stainings of TBX4 were almost at its peak (Fig. 3a, b). On E16, the rectum was completely separated from the urethra and the anal membrane ruptured. TBX4-immunopositive cells were also detected in the epithelium of distal rectum, but the number was significantly lower than that of E15 (Fig. 4a, b).

#### ARM group

On E13, the cloaca did not have the usual structure and no positive cells were seen in the hindgut and URS (Fig. 1c, d). On E14, the distance from the hindgut to the cloacal membrane was relatively long and the TBX4-immunopositive cells were scattered in the cloacal canal. Only a small number of positive cells were found in the hindgut near the cloacal canal. There are only very few positive cells in the most dorsal part of the cloacal membrane (Fig. 2c, d). On E15, a fistula is seen between the rectum and urethra. The expression of TBX4 in URS and rectal epithelium, fistula and dorsal cloacal membrane was very weak (Fig. 3c, d). On E16, the URS had not fused with the cloacal membrane and the anal membrane still existed. TBX4-immunopositive cells were scattered in the fistulous epithelium. There were only sporadic positive cells in the epithelium of the distal rectum (Fig. 4c, d).

### Western blot analysis

**Fig. 5:**
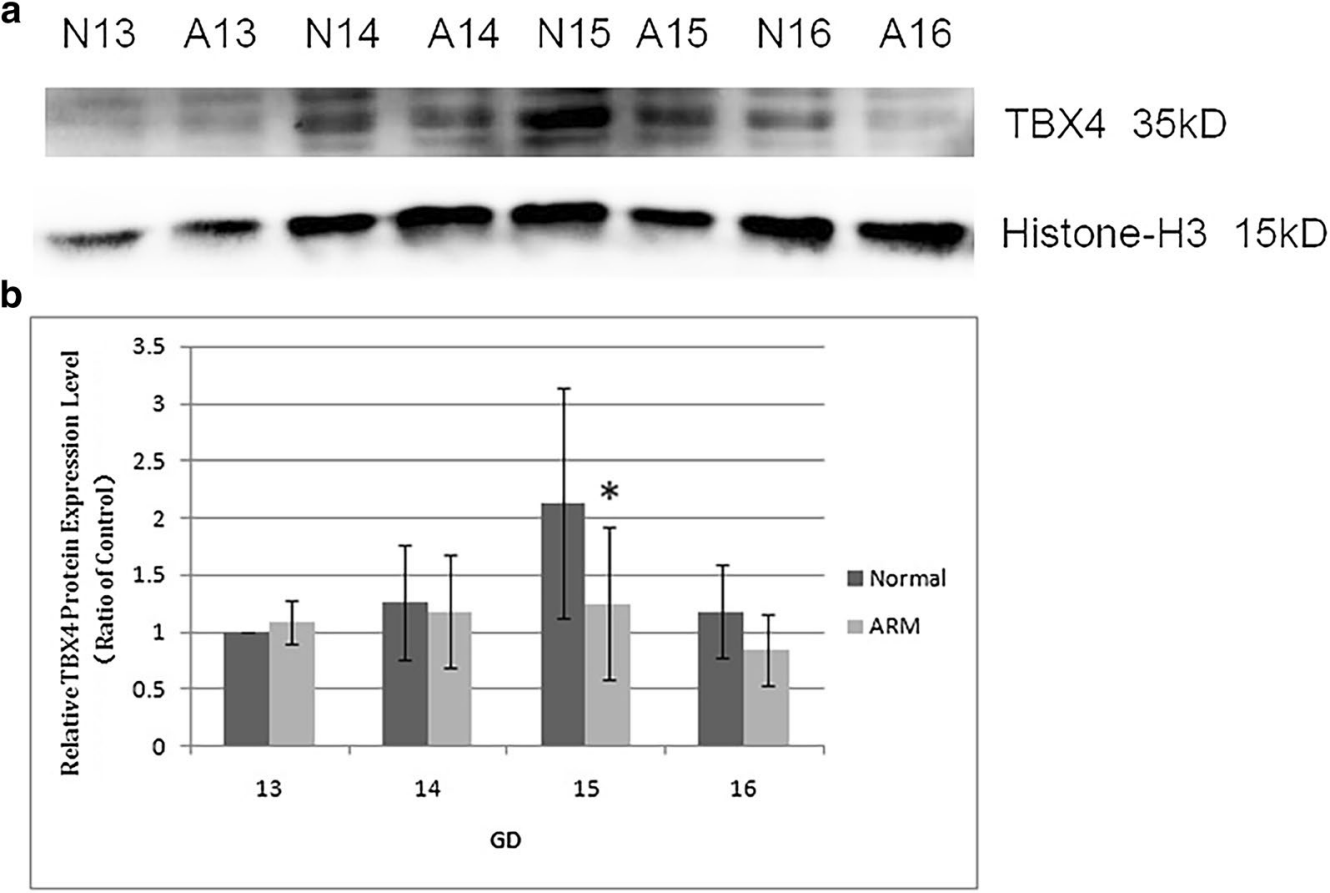
Western blot analyzed TBX4 protein level in the hindgut/rectum of the normal (N) and ARM (A) groups. TBX4 was detected as an approximately 35-kDa (kd) band on Western blots (**a**). Immunoblots showed a strong signal for TBX4 protein in the normal group, but a weak signal in the ARM group. Histone-H3 protein was used as an internal control. A histogram shows the trends of TBX4 expression at each time point (**b**). A peak can be noted on E15. Values are presented as mean ± SD. *Significant difference from the normal group

Western blotting was performed to quantify TBX4 protein expression in the hindgut/rectum during the anorectal development. TBX4 was detected as a band of about 35 kDa among the proteins extracted from both normal and ARM tissues. Each protein band was normalized to a corresponding Histone-H3 band (Fig. 5a). The results showed that TBX4 protein was expressed in both normal and ARM groups from E13 to E16 and TBX4 expression peaked on E15 in the normal group (Fig. 5b). As there was no significant difference in TBX4 protein expression on E13, E14 and E16 between the two groups, the TBX4 expression on E15 was significantly reduced in ARM group than normal group (E15 TBX4 protein: 1.25 ± 0.67 vs 2.13 ± 1.01, *P *= 0.021).

### Real-time RT-PCR

**Fig. 6:**
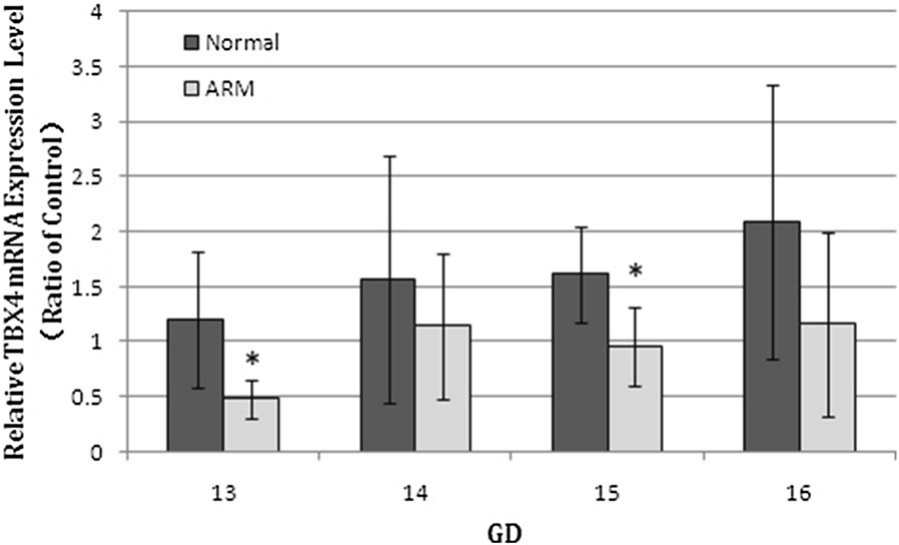
Real-time RT-PCR analyzed of TBX4 mRNA level in the hindgut/rectum of the normal (N) and ARM (A) groups. From E13 to E16, the expression of TBX4 mRNA gradually increased in the normal embryos. On E13 and E15, the expression of TBX4 mRNA significantly decreased in the hindgut/rectum of ARM embryos comparing with normal embryos. Values are presented as mean ± SD. *Significant difference from the normal group

The TBX4 mRNA expression level was assessed in the normal and ARM groups (Fig. 6). From E13 to E16, the expression of TBX4 mRNA gradually increased in the normal embryo. On E13 and E15, the mRNA expression of TBX4 significantly decreased in the hindgut/rectum of ARM rat embryos comparing with normal rat embryos (E13 TBX4 mRNA: 0.48 ± 0.18 vs 1.20 ± 0.62, *P *= 0.004; E15 TBX4 mRNA: 0.96 ± 0.35 vs 1.61 ± 0.44, *P *= 0.003).

## Discussion

Research on embryonic development of ARM requires the use of animal model because of the known difficulties in obtaining human embryos with ARM. About 80% offspring of the pregnant rats by intragastric ETU administration have ARM ranging from simple covered anus to recto-urethral fistula closely resembling those seen in neonates with ARM [15, 16]. ETU-induced rats are considered an ideal animal model to study the ARM embryogenesis and have been widely used [17–19].

ARM was a pathological process that caused by complex factors. Although there were heated debates between scholars who hold different views, it is generally believed that ARM was associated with dysplasia of cloaca and the failure of fusion of URS with the cloacal membrane [16, 20, 21]. The fusion of URS with the cloacal membrane is essential for the separation of rectum from urethra. The common canal between the rectum and urethra still exists, which leads to the rectourethral fistula or common cloaca.

Some scholars observed that the main body of URS curved ventrally behind the future bladder, whereas its epithelial tip deviated dorsally to fuse with the endodermal layer of the cloacal membrane at the location of the future anus on E15 in rat embryos. On E16, anal membrane ruptures and rectum communicates with the outside. In ETU-induced ARM rat embryos, the URS never fused with cloaca membrane and the dorsal cloacal membrane was maldeveloped.

In this study, we studied the spatial and temporal expression patterns of TBX4 during the anorectal development by immunohistochemical staining, Western blot analysis and real-time RT-PCR. The results of the immunohistochemistry showed that the expression of TBX4 had spatial specificity. TBX4-immunopositive cells were selectively expressed in the epithelium of hindgut/rectum, the URS and the dorsal cloacal membrane during the process of the anorectal morphogenesis. The expression of positive cells reached its peak on E15. However, cell expression was significantly reduced after anal membrane ruptured. Such a high specific TBX4 expression revealed that TBX4 may play a key role during the anorectal development.

In ARM group, the TBX4-immunopositive cells which were distributed in the epithelium of hindgut/rectum and URS were significantly less on E15. At the same time, the positive cells in the dorsal cloacal membrane were rare. These results were consistent with the Western and RT-PCR results. Their results showed that the expression level of TBX4 in normal embryos was higher, and the expression level of TBX4 in ARM embryos was significantly lower than that in normal embryos on E15. The down-regulation of TBX4 at key point of anorectal development might impact the fusion of the URS and the dorsal cloacal membrane and the rupture of the dorsal cloacal membrane, resulting in rectourethral fistula or persistent cloaca. Based on these findings, we propose that the down-regulation of TBX4 in ETU-induced rat embryos may lead to ARM.

TBX4 is encoded by the TBX4 gene located on human chromosome 17 [22], is a transcription factor that belongs to T-box gene family that control gene expression, participate in the regulation of embryonic development process. The T-box gene mutation is the cause of many human development syndromes [23–25]. TBX4 plays an important role in the development of the lungs, umbilicus and limb [26–28], but till now there is no research about the expression of TBX4 during the anorectal development in the normal and ARM embryos.

TBX4 is specific to induce the expression of FGF10 [11, 12]. FGF10 plays an important role in the survival and proliferation of intestinal epithelial cells [29]. Epithelial–mesenchymal action is crucial in the germination of the gastrointestinal tract. The FGF10−/− mutants fails to develop rectal epithelium, showing failure of union of the rectum and anus [10]. But the deformity is of a single type, it is presumed that isolated ARM in the absence of other anomalies may occur not as a result of deletion of the Fgf10 gene itself, but rather a mutation of the regulatory elements controlling Fgf10 expression in the rectum.

TBX4-FGF10 system controls the formation of lung buds and limb initiation during respiratory tract and limb embryonic development. Ectopic TBX4 induced ectopic bud formation in the esophagus by activating the expression of FGF10. Conversely, interference of TBX4 function resulted in repression of FGF10 expression and in failure of lung bud formation [11]. Karolak et al. studied the pathogenesis of interstitial neonatal lung disorders and identified concomitance of coding and non-coding single-nucleotide variants or copy-number variants involving TBX4 or FGF10 loci in lethal lung maldevelopment. They thought TBX4-FGF10 epithelial-mesenchymal signaling was very important in human lung organogenesis [30]. Animal studies had demonstrated that ablated TBX4 function during the hindlimb development suppressed FGF10 expression, indicating that FGF10 was likely a downstream target of TBX4 [12]. We hypothesized that TBX4-FGF10 signaling was involved in the rectal embryonic development and TBX4 may affect the development of the anorectum as a transcription factor by regulating the expression of FGF10.

## Conclusion

In conclusion, there was a significant difference in the expression of TBX4 between normal and ARM embryos during the development of anorectum. The results showed that the anorectal morphology may depend on the induction of TBX4 signal, and the down-regulation of TBX4 may be one of the reasons for ARM.

## Abbreviations

ARM: anorectal malformations
SHH: sonic hedgehog
BMP4: bone morphogenetic protein 4
FGF10: fibroblast growth factor 10
TBX4: T-box transcription factor 4
ETU: ethylenethiourea
IHC: immunohistochemistry
HRP: horseradish peroxidase
SDS-PAGE: sodium dodecyl sulfate-polyacrylamide gel electrophoresis
PVDF: polyvinylidene fluoride
cDNA: complementary DNA
URS: urorectal septum
UGS: urogenital sinus
